# Evidence of spatial clustering of childhood acute lymphoblastic leukemia cases in Greater Mexico City: report from the Mexican Inter-Institutional Group for the identification of the causes of childhood leukemia

**DOI:** 10.3389/fonc.2024.1304633

**Published:** 2024-02-14

**Authors:** David Aldebarán Duarte-Rodríguez, Janet Flores-Lujano, Richard J. Q. McNally, María Luisa Pérez-Saldivar, Elva Jiménez-Hernández, Jorge Alfonso Martín-Trejo, Laura Eugenia Espinoza-Hernández, Aurora Medina-Sanson, Rogelio Paredes-Aguilera, Laura Elizabeth Merino-Pasaye, Martha Margarita Velázquez-Aviña, José Refugio Torres-Nava, Rosa Martha Espinosa-Elizondo, Raquel Amador-Sánchez, Juan José Dosta-Herrera, Javier Anastacio Mondragón-García, Juana Esther González-Ulibarri, Sofía Irene Martínez-Silva, Gilberto Espinoza-Anrubio, María Minerva Paz-Bribiesca, Perla Salcedo-Lozada, Rodolfo Ángel Landa-García, Rosario Ramírez-Colorado, Luis Hernández-Mora, Marlene Santamaría-Ascencio, Anselmo López-Loyola, Arturo Hermilo Godoy-Esquivel, Luis Ramiro García-López, Alison Ireri Anguiano-Ávalos, Karina Mora-Rico, Alejandro Castañeda-Echevarría, Roberto Rodríguez-Jiménez, José Alberto Cibrian-Cruz, Karina Anastacia Solís-Labastida, Rocío Cárdenas-Cardos, Norma López-Santiago, Luz Victoria Flores-Villegas, José Gabriel Peñaloza-González, Ana Itamar González-Ávila, Martin Sánchez-Ruiz, Roberto Rivera-Luna, Luis Rodolfo Rodríguez-Villalobos, Francisco Hernández-Pérez, Jaime Ángel Olvera-Durán, Luis Rey García-Cortés, Minerva Mata-Rocha, Omar Alejandro Sepúlveda-Robles, Vilma Carolina Bekker-Méndez, Silvia Jiménez-Morales, Jorge Meléndez-Zajgla, Haydée Rosas-Vargas, Elizabeth Vega, Juan Carlos Núñez-Enríquez, Juan Manuel Mejía-Aranguré

**Affiliations:** ^1^ División de Desarrollo de la Investigación en Salud, Coordinación de Investigación en Salud, Centro Médico Nacional (CMN) “Siglo XXI”, Instituto Mexicano del Seguro Social (IMSS), Ciudad de Mexico, Mexico; ^2^ Unidad de Investigación Médica en Epidemiología Clínica, Unidad Médica de Alta Especialidad (UMAE), Hospital de Pediatría, Centro Médico Nacional (CMN) “Siglo XXI”, Instituto Mexicano del Seguro Social (IMSS), Ciudad de Mexico, Mexico; ^3^ Population Health Sciences Institute, Newcastle University, Royal Victoria Infirmary, Newcastle upon Tyne, United Kingdom; ^4^ Servicio de Oncología, Hospital Pediátrico “Moctezuma”, Secretaría de Salud de la Ciudad de Mexico (SEDESA), Ciudad de Mexico, Mexico; ^5^ Servicio de Hematología, Unidad Médica de Alta Especialidad Hospital de Pediatría, Centro Médico Nacional (CMN) “Siglo XXI”, Instituto Mexicano del Seguro Social (IMSS), Ciudad de Mexico, Mexico; ^6^ Departamento de Hemato-Oncología, Hospital Infantil de Mexico “Federico Gómez”, Secretaría de Salud (SS), Ciudad de Mexico, Mexico; ^7^ Servicio de Hematología, Instituto Nacional de Pediatría (INP), SS, Ciudad de Mexico, Mexico; ^8^ Servicio de Hematología Pediátrica, Centro Médico Nacional (CMN) “20 de Noviembre”, Instituto de Seguridad Social al Servicio de los Trabajadores del Estado (ISSSTE), Ciudad de Mexico, Mexico; ^9^ Servicio de Onco-Pediatría, Hospital Juárez de Mexico, SS, Instituto Politécnico Nacional 5160, Ciudad de Mexico, Mexico; ^10^ Servicio de Hematología Pediátrica, Hospital General de Mexico, SS, Ciudad de Mexico, Mexico; ^11^ Servicio de Hematología Pediátrica, Hospital General Regional (HGR) No 1 “Dr Carlos MacGregor Sánchez Navarro” Instituto Mexicano del Seguro Social (IMSS), Ciudad de Mexico, Mexico; ^12^ Servicio de Cirugía Pediátrica, Hospital General “Gaudencio González Garza”, Centro Médico Nacional (CMN) “La Raza”, Instituto Mexicano del Seguro Social (IMSS), Ciudad de Mexico, Mexico; ^13^ Servicio de Cirugía Pediátrica, Hospital General Regional (HGR) No 1 “Dr Carlos MacGregor Sánchez Navarro” Instituto Mexicano del Seguro Social (IMSS), Ciudad de Mexico, Mexico; ^14^ Hospital Pediátrico de Iztacalco, Secretaría de Salud de la Ciudad de Mexico (SEDESA), Ciudad de Mexico, Mexico; ^15^ Hospital Pediátrico de Iztapalapa, Secretaría de Salud de la Ciudad de Mexico (SEDESA), Ciudad de Mexico, Mexico; ^16^ Servicio de Pediatría, Hospital General Zona (HGZ) No 8 “Dr Gilberto Flores Izquierdo”, Instituto Mexicano del Seguro Social (IMSS), Ciudad de Mexico, Mexico; ^17^ Servicio de Pediatría, Hospital Juárez del Centro, SS, Cuauhtémoc, Ciudad de Mexico, Mexico; ^18^ Hospital General de Ecatepec “Las Américas”, Instituto de Salud del Estado de Mexico (ISEM), Ecatepec de Morelos, Mexico; ^19^ Hospital General “Dr. Manuel Gea González” SS, Ciudad de Mexico, Mexico; ^20^ Hospital Pediátrico “La Villa”, Secretaría de Salud de la Ciudad de Mexico (SEDESA), Ciudad de Mexico, Mexico; ^21^ Hospital Pediátrico “San Juan de Aragón”, Secretar´ıa de Salud de la Ciudad de Mexico (SEDESA), Ciudad de Mexico, Mexico; ^22^ Servicio de Pediatría, Hospital General Regional (HGR) No 72 “Lic. Vicente Santos Guajardo”, Instituto Mexicano del Seguro Social (IMSS), Tlalnepantla de Baz, Mexico; ^23^ Servicio de Cirugía Pediátrica, Hospital General Zona (HGZ) No. 32, Instituto Mexicano del Seguro Social (IMSS), Ciudad de Mexico, Mexico; ^24^ Servicio de Cirugía Pediátrica, Hospital Pediátrico de Moctezuma, Secretaría de Salud de la Ciudad de Mexico (SEDESA), Ciudad de Mexico, Mexico; ^25^ Servicio de Pediatría, Hospital Pediátrico de Tacubaya, Secretaría de Salud de la Ciudad de Mexico (SEDESA), Ciudad de Mexico, Mexico; ^26^ Urgencias Pediátricas, Hospital General Zona (HGZ) No 47, Instituto Mexicano del Seguro Social (IMSS), Ciudad de Mexico, Mexico; ^27^ Servicio de Cirugía Pediátrica, Hospital Regional “1° Octubre”, Instituto de Seguridad Social al Servicio de los Trabajadores del Estado (ISSSTE), Instituto Politécnico Nacional 1669, Revolución Instituto Mexicano del Seguro Social (IMSS), Ciudad de Mexico, Mexico; ^28^ Servicio de Pediatría, Hospital General Regional (HGR) No. 25 Instituto Mexicano del Seguro Social (IMSS), Ciudad de Mexico, Mexico; ^29^ Servicio de Pediatría, Hospital General de Zona con Medicina Familiar (HGZMF) No. 29, Instituto Mexicano del Seguro Social (IMSS), Ciudad de Mexico, Mexico; ^30^ Servicio de Cirugía Pediátrica, Hospital General Zona (HGZ) No. 27, Instituto Mexicano del Seguro Social (IMSS), Ciudad de Mexico, Mexico; ^31^ Servicio de Oncología, Instituto Nacional de Pediatr´ıa (INP), SS, Ciudad de Mexico, Mexico; ^32^ Delegación Regional Estado de Mexico Oriente, Instituto Mexicano del Seguro Social (IMSS), Naucalpan de Juárez, Mexico; ^33^ Laboratorio de Biología Molecular, Unidad Me´dica de Alta Especialidad (UMAE), Hospital de Pediatría, Centro Me´dico Nacional (CMN) “Siglo XXI”, Instituto Mexicano del Seguro Social (IMSS), Ciudad de Mexico, Mexico; ^34^ Unidad de Investigación Médica en Inmunología e Infectología, Hospital de Infectología “Dr Daniel Méndez Hernández”, Centro Me´dico Nacional (CMN) “La Raza”, Instituto Mexicano del Seguro Social (IMSS), Ciudad de Mexico, Mexico; ^35^ Laboratory of Innovation and Precision Medicine, Nucleus A. Instituto Nacional de Medicina Genómica (INMEGEN), Ciudad de Mexico, Mexico; ^36^ Laboratorio de Genómica Funcional del Cáncer, Instituto Nacional de Medicina Genómica (INMEGEN), Ciudad de Mexico, Mexico; ^37^ Unidad de Investigación Médica en Genética Humana, Unidad Me´dica de Alta Especialidad (UMAE), Hospital de Pediatría, Centro Me´dico Nacional (CMN) “Siglo XXI”, Instituto Mexicano del Seguro Social (IMSS), Ciudad de Mexico, Mexico; ^38^ Instituto de Ciencias de la Atmósfera y Cambio Climático, Universidad Nacional Autónoma de Mexico (UNAM), Ciudad de Mexico, Mexico; ^39^ División de Investigación en Salud, Unidad Me´dica de Alta Especialidad (UMAE) Hospital de Pediatría “Dr. Silvestre Frenk Freund”, Centro Me´dico Nacional (CMN) “Siglo XXI”, Instituto Mexicano del Seguro Social (IMSS), Ciudad de Mexico, Mexico; ^40^ Facultad de Medicina, Universidad Nacional Autónoma de Mexico (UNAM), Ciudad de Mexico, Mexico

**Keywords:** leukemia, child, spatial clustering, SaTScan software to analyze spatial, electromagnetic fields, environmental exposure, urban population, developing countries

## Abstract

**Background:**

A heterogeneous geographic distribution of childhood acute lymphoblastic leukemia (ALL) cases has been described, possibly, related to the presence of different environmental factors. The aim of the present study was to explore the geographical distribution of childhood ALL cases in Greater Mexico City (GMC).

**Methods:**

A population-based case-control study was conducted. Children <18 years old, newly diagnosed with ALL and residents of GMC were included. Controls were patients without leukemia recruited from second-level public hospitals, frequency-matched by sex, age, and health institution with the cases. The residence address where the patients lived during the last year before diagnosis (cases) or the interview (controls) was used for geolocation. Kulldorff’s spatial scan statistic was used to detect spatial clusters (SCs). Relative risks (RR), associated p-value and number of cases included for each cluster were obtained.

**Results:**

A total of 1054 cases with ALL were analyzed. Of these, 408 (38.7%) were distributed across eight SCs detected. A relative risk of 1.61 (p<0.0001) was observed for the main cluster. Similar results were noted for the remaining seven ones. Additionally, a proximity between SCs, electrical installations and petrochemical facilities was observed.

**Conclusions:**

The identification of SCs in certain regions of GMC suggest the possible role of environmental factors in the etiology of childhood ALL.

## 1 Introduction

The frequency of childhood acute leukemias (AL) in Mexico City has been reported to be amongst the highest in the world, mainly, for the acute lymphoblastic leukemia (ALL) subtype (1–3). The etiology of AL remains unclear in most cases. It seems to be the result from an interaction between genetic susceptibility and exposure to environmental factors (4; 5–7).

The spatial analysis of disease incidence distribution has been acknowledged as a valuable approach for uncovering essential insights into the etiology of a disease. (8, 9). In Mexico, there have been limited spatial analysis studies conducted to date related to childhood leukemia. In a preliminary report, a significant spatial cluster (SC) of childhood ALL cases was detected at the eastern side of Mexico City (10). In another research, conducted in the city of Guadalajara, three SCs of ALL cases were also described (11).

**Figure 1:**
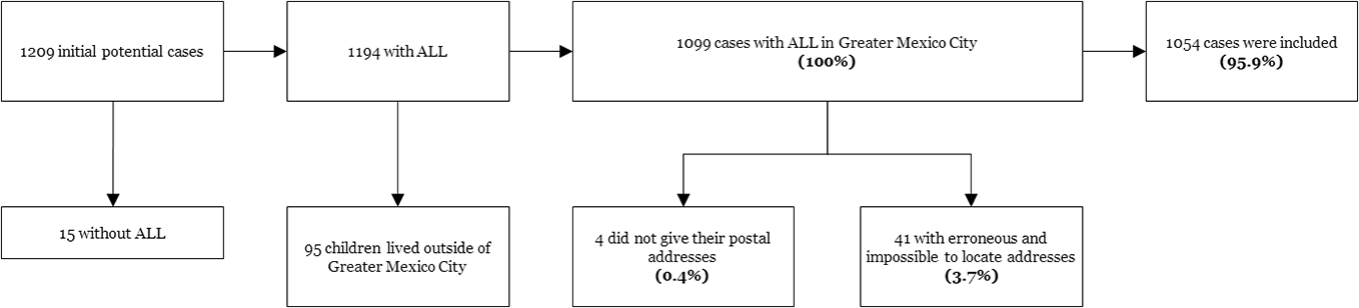
Geographic stratification of Greater Mexico City.

On the other hand, in a recent investigation conducted in Mexico City, AL incidence rates displayed differences among municipalities suggesting a potential heterogeneous geographical distribution (3). Noteworthy, Mexico City and its surrounding metropolitan area [also known as the Greater Mexico City (GMC)] has seventy-five municipalities being one of the largest urban agglomerations globally. The core of the metropolis is a proper urban area whereas the outer is considered as a rural–urban fringe area (see **Figure 1**). When these areas are well-delimited they may significantly differ in demographic factors such as the population density, the main economic activities, exposure to environmental hazards among other which could have an impact in the incidence of childhood leukemia (12–14).

Several research studies have highlighted the potential associations between the exposure to environmental factors and the development of leukemia in the pediatric population of GMC. These factors include the exposure to extremely-low-frequency magnetic fields (ELF-MFs) (Juan C. 15, 16), the maternal and paternal ages at conception of the index child (17), a greater child´s birthweight (18), viral infections (19), father’s occupational exposure (20, 21), allergies (22), breastfeeding (J 23), and early-life infections (23). Additionally, the relationship between genetic and environment interactions has been explored. Particularly, for the exposure to fertilizers, insecticides, hydrocarbon derivatives and parental tobacco smoking (24).

The aim of the present study was to explore the geographical distribution of childhood ALL cases in GMC, a region characterized by a high incidence of the disease.

## 2 Methods

### 2.1 Population

A population-based case-control study was conducted. Children <18 years old, newly diagnosed with ALL and GMC residents represented the group of cases. They were recruited from public hospitals where it has been estimated that 97.5% of children with leukemia from GMC are attended (25). Case registration required that trained personnel were assigned to each participating hospital to identify incident cases of leukemia through reviews of clinical charts. Afterwards, parents were approached and invited to participate. Given that careful case registration is essential for successful conduct of case-control studies, we followed the recommendations of the IARC for the planning and development of population-based cancer registries (26).

ALL diagnosis was established based on clinical features, and bone marrow aspirate findings, including cell morphology, immunophenotype, and genetics, as defined in 2008 by the World Health Organization (WHO) for the classification of lymphoid neoplasms.

The controls were selected from second-level hospitals of the same health institution that referred the children with ALL to the third-level care hospitals. The controls were children without leukemia who were treated at different hospital departments, such as ambulatory surgery, pediatrics, orthopedic outpatient clinics and the emergency room. Children with diagnoses of neoplasms, hematological diseases, allergies, infections, and congenital malformations were not selected as controls. A frequency-matched approach was used between cases and controls according to the following variables: child´s sex, age (at diagnosis for cases, and at the time of the interview for the controls) and health institution. Age was estimated in months, with a difference between cases and controls no greater than 12 months.

There were two different periods for the ascertainment of cases and controls: Cases (Period 1: January 1, 2006, to December 31, 2007; Period 2: January 1, 2010, to December 31, 2012); Controls (Period 1: January 1, 2000 to December 31, 2007; Period 2: January 1, 2010 to December 31, 2013).

### 2.2 Data collection

Data collection was obtained by trained personnel through the revision of clinical charts and in-person interviews with the parents or guardians of the cases and controls (21) using a previously standardized questionnaire(J 23). The two periods for case ascertainment represent the complete years when sufficient financial support was available for conducting the interviews, clinical charts revisions and all the procedures required for the present research. Therefore, a representative sample of the incident cases with ALL diagnosed during those years in GMC was included. On the other hand, the control recruitment period started six years prior to the inclusion of cases and concluded one year after the end of the case ascertainment period. This allowed us for achieving a larger control pool for selecting the controls who had complete geolocation data and fulfilled the matching criteria.

Information recorded included: the postal addresses where the child lived the last year before the diagnosis (for cases) or at the moment of the interview (for controls). Additionally, random cross-checking telephone calls were performed by the supervisor of the personnel to ensure the accuracy of the information.

### 2.3 Geolocation of cases and controls, and study area

The street centroid was used for georeferencing the postal addresses, taking as the reference the intersection between the two closest streets where the child lived. Cartographic information was obtained through the country’s National Institute of Statistics and Geography (INEGI) information reported for 2010 (27) and by using Google Maps.

However, the information on postal addresses was partially obtained from the participants due to the following reasons: a) they felt distrustful, b) they did not know the postal address accurately, and c) they provided an address which differs from the officially recorded. In these situations, the neighborhood centroid strategy developed by Freire de Carvalho was followed (28).

When it was not possible to obtain the minimal information needed to geolocate or when the parents or guardians explicitly refused to provide their addresses, the individuals were excluded from the analysis.

Afterwards, Greater Mexico City was stratified into smaller spatial units: in order to differentiate between areas with different population density, the most urbanized part of the metropolis was classified as the urban area, whereas, the most external and least urbanized areas that are still quite rural were classified as rural–urban fringe area (see **Figure 1**), based on Duhau and Giglia (29). All the data were mapped and, to ensure the anonymity and confidentiality of the individuals participating in this work, none of the exhibited maps represent the children’s precise addresses so that they cannot be identified.

### 2.4 Spatial scan statistic

The spatial scan statistic proposed by Kulldorff (30, 31) using the SaTScan™ software was employed (Martin Kulldorff, Harvard Medical School and Harvard Pilgrim Health Care Institute, Boston, USA, https://www.satscan.org/). The probability model of Bernoulli was selected as it has been previously used in other studies on childhood leukemia (32, 33). Some of the advantages of this probability model are: 1) it is appropriate for detecting spatial clusters using case-control data (34); 2) it eliminates the disadvantage of studying areas with different population densities, as in Greater Mexico City; 3) it can control for covariates and 4) it controls for issues related to multiple testing (35). Inclusion of covariates allowed analysis of differences between urban and rural-urban fringe areas.

In addition, the method has been described as a scanning of the study area using a window of geometric exploration (36). This window is virtual and can take a circular or ellipsoidal geometric shape. Particularly, we chose for the circular shapes windows taking into account that the urban core of Greater Mexico City has the same length from north to south as from east to west. For each window location and size, the SaTScan calculates the number of observed and expected observations inside the window (37). Then, a relative risk (RR) was estimated for each childhood ALL cluster being the RR interpreted as the ratio of the probability of being within the cluster versus the risk of being outside the cluster. Additionally, the SaTScan Bernoulli model uses a log likelihood ratio test of the probability (LLR) which allows for identifying the main SC by selecting the circular window with the maximum LLR. To assess the precision and statistical significance of the findings, SaTScan simulates many random datasets to construct a distribution of geographic points that satisfies the assumptions of the null hypothesis (no clustering). The precision (p value) was assessed using 99999 simulations using the Monte Carlo hypothesis test. A p-value less than 0.05 was considered statistically significant.

In the present study, only the non-overlapping SCs with the largest LLRs, statistically significant (<0.05), with at least ten cases per cluster were reported. This last criterion was considered because few cases with ALL were included in the remaining detected SCs. This small number would hamper any epidemiological interpretation of findings as it represents less than 1% of the population of cases analyzed (n=1,054).

## 3 Results

**Figure 2:**
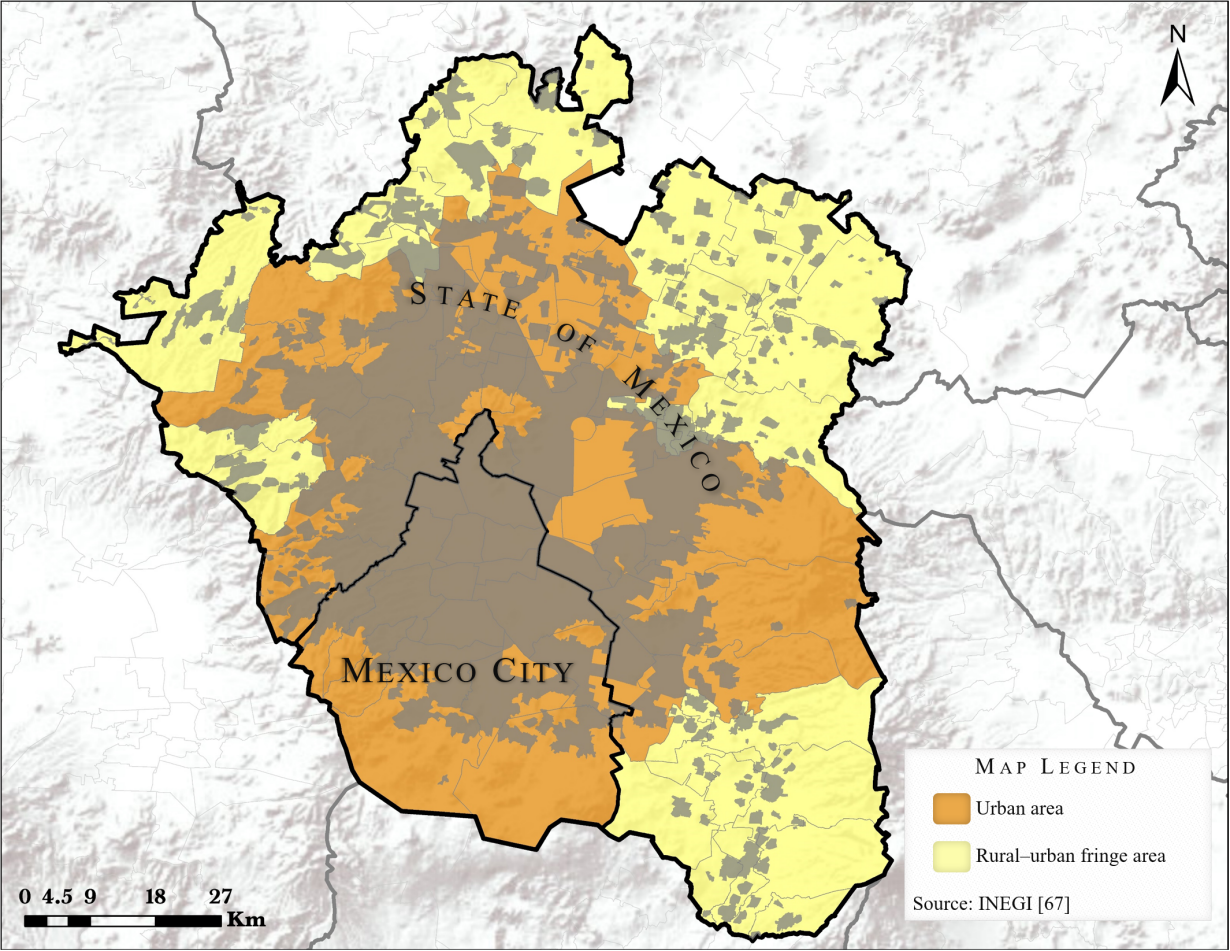
Selection of cases included in the study.

A total of 1209 cases with childhood ALL were diagnosed during the study period. Of these, 1054 (95.9%) cases fulfilled the selection criteria and were included in the study (see **Figure 2**). The predominant sex of cases was male, accounting for 53% (n=559). The cases were distributed across the following age groups: <1 year: 38 (3.6%); 1-4 years: 417 (39.5%); 5-9 years: 305 (28.9%); 10-14: 234 (22.2%) and 15-17 years: 60 (5.6%). According to the morphological features of the malignant cells, patients with ALL were classified as: L1 (n= 708; 67.2%); L2 (n= 329; 31.2%); and L3 (n= 17; 1.6%). No statistically significant differences were observed between cases and controls according to sex, age and health institution (see **Table 1**: Demographic and other characteristics of cases and controls included in the present study).

**Table 1:** Demographic and other characteristics of cases and controls included in the present study.

Variable	Cases	Controls	p value*
	n = 1054	n= 1054	
	n (%)	n(%)	
Sex			
Male	559 (53)	559 (53)	1.00
Female	495 (47)	495 (47)	
Age group (years)			
<1	38 (3.6)	37 (3.5)	0.99
1-4	417 (39.5)	413 (39.2)	
5-9	305 (28.9)	312 (29.6)	
10-14	234 (22.2)	232 (22)	
15-17	60 (5.6)	60 (5.7)	
Health Institution			
IMSS	441 (42)	438 (41.6)	0.99
Minister of Health	581 (55)	584 (55.4)	
ISSSTE	32 (3)	32 (3)	

*Chi-square test.

IMSS, Instituto Mexicano del Seguro Social; ISSSTE, Instituto de Seguridad Social al Servicio de los Trabajadores del Estado.

**Figure 3:**
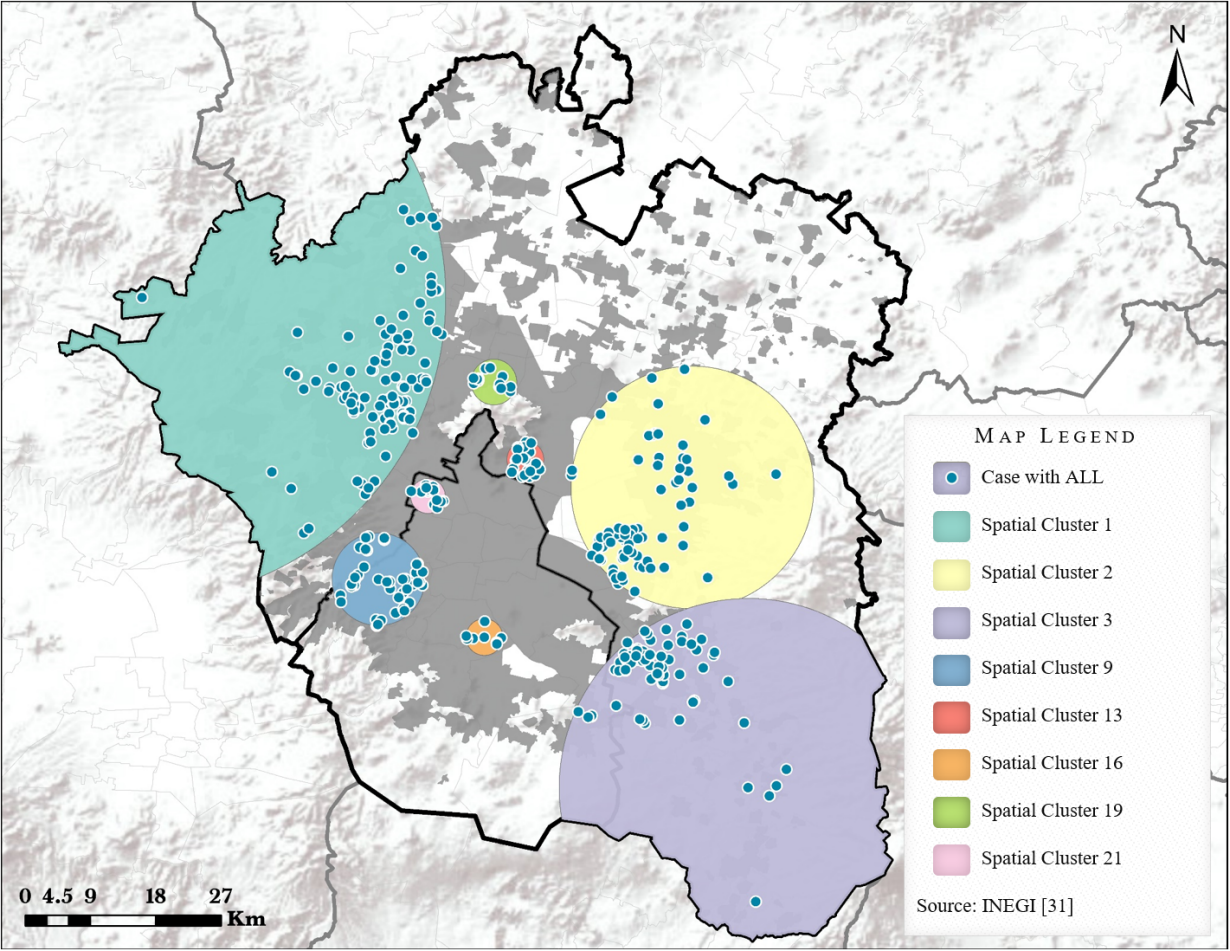
Spatial clusters of ALL cases in Greater Mexico City.

In the present research, eight SCs of cases with ALL were found in Greater Mexico City (see **Figure 3**). The sum of cases with ALL within the eight clusters represented 38.7% (n=408) of the total children included. The main cluster (SC #1) had an LLR=15,317.30, a RR=1.61 and a p-value <0.0001. It also included the largest number of cases (n=132) in comparison to the other clusters with a radius greater than 40 km. The other SCs included the following number of cases: #2 (n=91); #3 (n=69); #9 (n=48); #13 (n=29); #16 (n=11); #19 (n=14) and the cluster #21 included 14 cases. Similar LLRs, RRs, and p-values were noted for these SCs (see **Supplementary Table 1**: Characteristics of spatial clusters of children with ALL identified in Greater Mexico City). When differences were examined based on study areas, no significant distinctions were observed between urban and rural-urban fringe areas.

**Figure 4:**
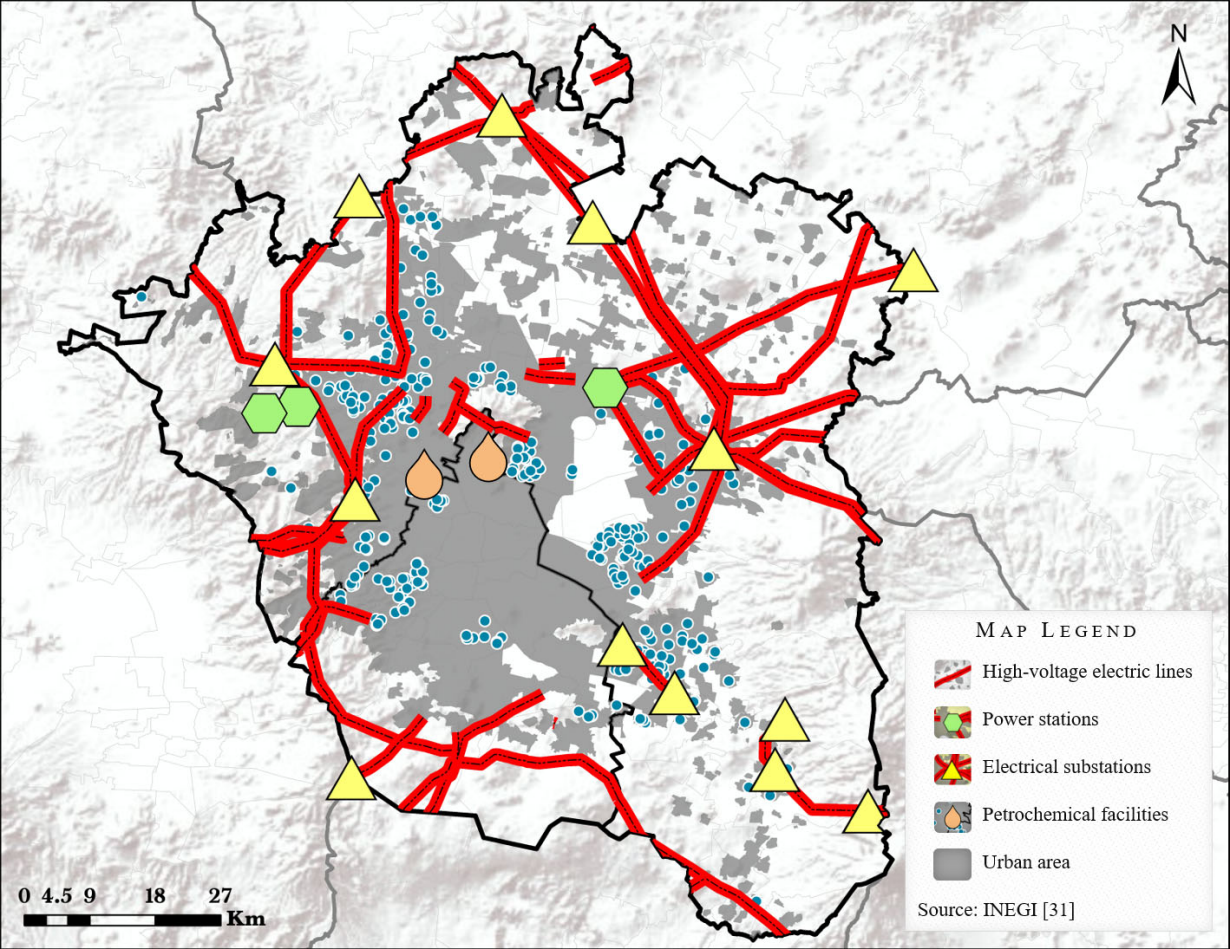
High-voltage electric and petrochemical installations in Greater Mexico City.

Notably, six out of eight SCs were closed to high−voltage electric lines and high−voltage electric installations (**Figure 4**). Furthermore, it was also noted that the remaining two SCs were in proximity to areas where former petrochemical industrial facilities had been located (closed a decade before the beginning of the present study). One of these facilities, was the former Azcapotzalco Refinery, and the other, was the San Juan Ixhuatepec petrochemical storage and distribution plant.

## 4 Discussion

In this study, a heterogeneous spatial distribution of children with ALL living GMC was identified. Additionally, eight SCs of children with ALL in Greater Mexico City were detected using the Kulldorff’s spatial scan statistics.

To our knowledge, the present work is one of the few studies conducted in a city from a developing country aimed to investigate the spatial distribution of pediatric cases with ALL (38). The vast majority of these types of analyses have been carried out in populations from developed countries with larger and very affluent geographic areas―like European whole countries―(39–41). The disadvantages of studying greater geographical areas for identifying SCs have been explained (42). In addition, it has been suggested that the best scale of geographic analysis to identify SCs is the small-scale, or when the territory has a high population density, such as is the case of GMC (42–44).

The identification of significant SCs of childhood ALL cases in the present research supports to various hypotheses regarding risk factors potentially implicated in the development of this neoplasm. These hypotheses include the association with identifiable sources of exposure to harmful environmental agents (such as pesticides, insecticides, etc.), the possibility of an infectious etiology, among other factors (8, 9). We did not observe differences in the SCs distribution between urban and rural−urban fringe areas, as it has been reported in other investigations (14, 45–47). One possible reason for this negative result could be the minimal differences between the studied areas regarding factors such as: the population density, demographics, education, lifestyle, economic activities, transportation, day-to-day activity, among others. All these, as a consequence of the metropolitan system dynamics of GMC which tends to homogenize the distribution of these factors between regions. Therefore, it is likely that the differences between urban and rural−urban fringe areas were so small that they could not be detected by our methodological approach.

Interestingly, most of the SCs detected in the present study were closed to electrical installations whereas other SCs were in proximity to potential sources of hydrocarbons (former petrochemical facilities).

Firstly, the association between living near to high−voltage transmission lines and the risk of childhood AL has been explored in different populations (48, 49). The mechanisms that could explain this association are related with the exposure to the generated extremely-low-frequency magnetic fields (ELF-MFs) and the ionized particles of air produced by corona discharge (50) which has been suggested as a possible explanation of the high incidence rates of childhood AL in Mexico City (51).

Specifically, the association between ELF-MFs and the risk of childhood ALL development has been reported in different studies conducted in Mexico City using direct or indirect methods for assessing the exposure. Particularly, a high frequency of exposure to increased levels of ELF-MFs has been reported in our population. Moreover, an association between ELF-MFs and the risk of childhood AL has been identified in children from Mexico City, a finding that has also been reported in other populations (15, 16).

On the other hand, the relationship between exposure to derivatives from the petrochemical industrial activity and risk of childhood leukemia has also been documented (52–54). Notably, in a study conducted in Mexico City it was reported that the interaction between hydrocarbon exposure and genetic polymorphisms of *NAT2* is associated with a high risk of developing childhood ALL. (24). However, these hypotheses require further study.

## 5 Study limitations

A possible limitation of the present investigation was the fact that a hospital-based recruitment of controls was followed instead of a random recruitment of controls from the source population, which has been recommended for this type of studies (55, 56). Nevertheless, if this last strategy had been implemented, it could have generated a low participation rate and a high cost, which exceeded our budget. Another limitation was produced by the difference in the periods of recruitment of cases and controls which restricted the analysis to a spatial clustering approach instead of a space-time cluster analysis that could have provided more insights about the effect of environmental factors occurring at specific times and places; an example of this would be the ability to detect patterns of childhood ALL incidence in relation to the date of birth of the children, or in relation to the time of diagnosis of the disease. On the other hand, exploring the geographic distribution of ALL cases among different age groups would be interesting, given the variation in disease incidence across age groups. However, the limited sample size hinders the feasibility of conducting a stratified analysis with sufficient statistical power. Additionally, it is important to continue the study of the geographical distribution of childhood ALL cases by analyzing updated geolocation data considering the persistently high incidence rates of this neoplasm in GMC. Lastly, we also reiterate the relevance of developing and/or consolidating cancer registries as the base to conduct studies for identifying SCs and risk factors associated with the development of ALL in the pediatric population.

## 6 Conclusions

The geographical distribution of childhood ALL cases in Greater Mexico City was heterogeneous across the territory of the metropolis. The identification of spatial clusters in certain regions of GMC suggest the possible role of environmental factors in the etiology of the disease. However, further investigations are required to elucidate the environmental hazards associated.

## Data availability statement

The raw data supporting the conclusions of this article will be made available by the authors, without undue reservation.

## Ethics statement

The studies involving humans were approved by National Scientific Research and Ethics Committee of the Mexican Institute of Social Security. The studies were conducted in accordance with the local legislation and institutional requirements. Written informed consent for participation in this study was provided by the participants’ legal guardians/next of kin.

## Author contributions

DD-R: Formal analysis, Investigation, Writing – review & editing, Conceptualization, Methodology, Software, Visualization, Writing – original draft. JF-L: Formal analysis, Investigation, Data curation, Validation, Writing – original draft. RM: Formal analysis, Conceptualization, Methodology, Software, Supervision, Writing – review & editing. MP-S: Writing – review & editing, Data curation, Investigation, Resources. EJ-H: Investigation, Resources, Writing – review & editing. JM-T: Resources, Writing – review & editing. LE-H: Resources, Writing – review & editing. AM-S: Resources, Writing – review & editing. RP-A: Resources, Writing – review & editing. LM-P: Resources, Writing – review & editing. MV-A: Resources, Writing – review & editing. JT-N: Resources, Writing – review & editing. RE-E: Resources, Writing – review & editing. RA-S: Resources, Writing – review & editing. JD-H: Resources, Writing – review & editing. JM-G: Resources, Writing – review & editing. JG-U: Resources, Writing – review & editing. SM-S: Resources, Writing – review & editing. GE-A: Resources, Writing – review & editing. MP-B: Resources, Writing – review & editing. PS-L: Resources, Writing – review & editing. RL-G: Resources, Writing – review & editing. RR-C: Resources, Writing – review & editing. LH-M: Resources, Writing – review & editing. MS-A: Resources, Writing – review & editing. AL-L: Resources, Writing – review & editing. AG-E: Resources, Writing – review & editing. LG-L: Resources, Writing – review & editing. PS-L: Resources, Writing – review & editing. A-AÁ: . KM-R: Resources, Writing – review & editing. AC-E: Resources, Writing – review & editing. RR-J: Resources, Writing – review & editing. JC-C: Resources, Writing – review & editing. KS-L: Resources, Writing – review & editing. RC-C: Resources, Writing – review & editing. NL-S: Resources, Writing – review & editing. LF-V: Resources, Writing – review & editing. JP-G: Resources, Writing – review & editing. AG-Á: Resources, Writing – review & editing. MS-R: Resources, Writing – review & editing. RR-L: Resources, Writing – review & editing. LR-V: . FH-P: Resources, Writing – review & editing. JO-D: Resources, Writing – review & editing. LG-C: Resources, Writing – review & editing. MM-R: Resources, Writing – review & editing. OS-R: Resources, Writing – review & editing. VB-M: Resources, Writing – review & editing. SJ-M: Resources, Writing – review & editing. JM-Z: Resources, Writing – review & editing. HR-V: Resources, Writing – review & editing. EV: Resources, Writing – review & editing. JN-E: Resources, Writing – review & editing, Formal analysis, Investigation, Project administration. JM-A: Conceptualization, Funding acquisition, Investigation, Methodology, Writing – review & editing, Project administration, Supervision.
